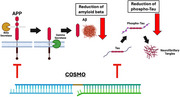# Simultaneous reduction of amyloid beta and phosphorylated tau for Alzheimer’s Disease with Chemically Optimized Stringed Modifiable Oligonucleotides (COSMOs)

**DOI:** 10.1002/alz70859_103402

**Published:** 2025-12-25

**Authors:** Jack Stahl, Aswathy Joji, Elianny Perozo Rojas, Olga Khorkova, Claude‐Henry Volmar, Claes Wahlestedt

**Affiliations:** ^1^ University of Miami Miller School of Medicine, Center for Therapeutic Innovation, Miami, FL USA

## Abstract

**Background:**

Alzheimer’s Disease (AD) is defined by deposition of amyloid beta (A𝛣), phosphorylated tau (p‐Tau), and neurodegeneration. Several A𝛣‐targeting antibodies have been approved for AD in recent years, but cause swelling/hemorrhaging of the brain and fail to reduce p‐Tau, which drives disease progression. Oligonucleotide therapeutic approaches have recently shown promise and have been well tolerated in early‐stage clinical trials, with an amyloid precursor protein (APP) siRNA reducing amyloid beta and a microtubule‐associated protein tau (MAPT) ASO reducing p‐Tau. We have developed a novel class of multi‐targeting siRNAs called chemically optimized stringed modifiable oligonucleotides (COSMOs) which are capable of simultaneously targeting APP and MAPT to reduce both A𝛣 and p‐Tau.

**Method:**

Unique siRNAs targeting APP or MAPT were designed, synthesized, and screened to select top hits for COSMO synthesis. Human SK‐N‐AS cells were transfected with COSMOs as well as individual siRNAs for 2‐5 days prior to quantifying mRNA levels of APP and MAPT with qPCR as well as protein levels of APP, Tau, A𝛣42, and p‐Tau181 with ELISA. Cellular viability following transfection was determined with CellTiter Glo. COSMOs were incubated with cellular lysates from SK‐N‐AS cells for 72 hours at 37C prior to running gel electrophoresis to determine COSMO cleavability.

**Result:**

We first found that our top APP‐ and MAPT‐targeting siRNAs are active at picomolar concentrations in vitro. We then confirmed that co‐transfection of our top siRNAs simultaneously reduces both A𝛣 and p‐Tau. Following synthesis and transfection of APP‐ and MAPT‐targeting COSMOs, we observed simultaneous reductions of APP, Tau, A𝛣, and p‐Tau protein at picomolar concentrations. We also show that APP‐ and MAPT‐targeting COSMOs are less toxic than individual siRNAs in vitro. Lastly, COSMOs can be engineered to be either cleavable or non‐cleavable for optimal pharmacokinetics and pharmacodynamics.

**Conclusion:**

To date, there are no approved therapeutics for AD capable of reducing both A𝛣 and p‐Tau. Oligonucleotide therapeutics for AD have shown early clinical success in reducing either A𝛣 or p‐Tau, but not both. COSMOs are a promising new class of therapeutics capable of reducing both A𝛣 and p‐Tau for AD.